# Optimizing trap design, lure, and color for monitoring *Chrysobothris mali* (Coleoptera: Buprestidae) in California walnut orchards

**DOI:** 10.1093/ee/nvag033

**Published:** 2026-04-16

**Authors:** Jhalendra Rijal, Samaneh Sakaki, Sudan Gyawaly, Karla Addesso

**Affiliations:** University of California Agriculture and Natural Resources, UC Statewide IPM Program, Davis, CA, USA; University of California Agriculture and Natural Resources, UC Statewide IPM Program, Davis, CA, USA; University of California Agriculture and Natural Resources, UC Statewide IPM Program, Davis, CA, USA; Department of Agricultural and Environmental Sciences, Otis L. Floyd Nursery Research Center, Tennessee State University, McMinnville, TN, USA

**Keywords:** Pacific flatheaded borer, walnut, trapping, Buprestidae, early detection

## Abstract

Pacific flatheaded borer, *Chrysobothris mali* (Coleoptera: Buprestidae), is a reemerging pest of walnuts in California. With the expansion of walnut production acres and increased incidences of severe droughts that stress orchards, *C. mali* has gained economically important pest status in recent years. In this article, we explored different trap designs, colors, and lures for early detection and monitoring of *C. mali* in commercial walnut orchards in California. In multi-year studies, ground-installed 4-foot-tall purple triangular traps with adhesives on the outer surface were more attractive than the purple panel sticky traps with or without volatile lures. Furthermore, among multiple colors of triangular traps, yellow and red traps captured more *C. mali* adults compared to other colors, including purple, in replicated trials conducted in 2 walnut orchards. The yellow triangular trap captured adults in these walnut orchards most consistently and earlier in the season. The utility of these traps in monitoring *C. mali* in walnut orchards and future directions of monitoring research are discussed.

## Introduction

Tree nut production, including English walnut (*Juglans regia*), is among California’s most important agricultural industries (Liang et al. 2024). As the leading producer of walnuts in the United States, California accounts for nearly 100% of domestic production (CDFA 2023) and 26% of global exports, with a farmgate value of ∼1.3 billion US dollars (CDFA 2023, INC 2023). Walnuts are cultivated on approximately 150,000 hectares in California, producing 607,814 tons of marketable nuts annually (CDFA 2023). With its large production scale and global reach, walnut is an important agricultural commodity and a significant contributor to the state’s economy (Woolwine et al. 2020, Liang et al. 2021).

Insect damage is a major cause of revenue loss in tree crops, including walnuts. Multiple insect species, such as navel orangeworm (*Amyelois transitella*), codling moth (*Cydia pomonella*), and walnut husk fly (*Rhagoletis completa*), attack walnut fruits and cause economic damage by reducing the yield and quality (Grant et al. 2017). Indirect pests include multiple species of mites, scales, aphids, and wood-boring beetles. Wood boring buprestids attack live plant tissues, mainly weakened parts such as diseased or sunburnt branches or trunks. Adult females of these beetles detect stressed trees, including those water-stressed (Evans et al. 2004), for oviposition. The larvae, also known as flatheaded borers, feed on the cambium tissue at vulnerable spots on the plant’s roots, trunks, and branches, causing tree decline over time (Burke 1917).

Flatheaded borers are critical pests of several tree species due to the severe damage they can cause and the challenges associated with their effective control (Adkins et al. 2009). In recent years, the Pacific flatheaded borer, *Chrysobothris mali* Horn (Coleoptera: Buprestidae), has become an economically important pest of nursery, landscape, and tree nut crops, potentially due to severe drought (Seagraves et al. 2013, Wiman et al. 2019, Addesso et al. 2020, Rijal 2020). *Chrysobothris mali* is native to the western United States and has long been recognized as a pest of newly established orchards, shade trees, and certain forest tree species (Burke 1917, Burke 1919, Burke and Boving 1929). In the late 1960s, *C. mali* was considered a significant threat to orchard crops in California’s Central Valley (Davis et al. 1968, McNelly et al. 1969). Although *C. mali* has been reported as an occasional pest of young fruit and nut crops in the past, it has become widespread in walnut orchards in recent years, affecting young, mature, and healthy trees, and has reached economic pest status (Rijal and Seybold 2019a). While some studies have addressed *Chrysobothris* species in tree crops, very little research has specifically examined *Chrysobothris mali* under California conditions in recent decades.

*Chrysobothris mali* is a reemerging pest affecting nursery, landscape, and tree nut crops throughout the United States (Seagraves et al. 2013, Wiman et al. 2019, Rijal and Seybold 2019a, Addesso et al. 2020). In recent years, damage caused by *C. mali* has increased in commercial tree crops across the western United States (Dawadi et al. 2019, Rijal and Seybold 2019b, Wiman et al. 2019, Addesso et al. 2020, Rudolf and Wiman 2023). In California, *C. mali* has become a significant pest in walnut orchards since the late 2010s (Rijal and Seybold 2019a). Adult emergence in California walnuts begins as early as April and can last until July and August (Burke 1929, Gyawaly and Rijal 2025). Adult females deposit eggs singly within the crevices of the bark on host trees (Burke 1929). Neonatal larvae chew through the egg chorion, enter the bark through cracks and openings, and initially feed under the bark. Mature larvae (ie prepupal stage) make a pupation chamber in the center of the pith for overwintering and subsequently undergo pupation in the spring (Sakaki et al., unpublished data). The damage potential of the borer is enhanced by its ability to travel inconspicuously and securely within woody materials. Currently, the most effective and recommended practice for reducing the pest population in walnuts is winter pruning of infested branches (Grant et al. 2017).

Integrated pest management (IPM) of any insect pest starts with effective detection and monitoring tools. Numerous studies have investigated the effectiveness of traps with varying colors and shapes for monitoring or mass-trapping of this group of insects in forest systems (Coleman et al. 2014, Poland et al. 2019, Cavaletto et al. 2020, Kuhn et al. 2024). In the *Chrysobothris* genus, particularly the *C. femorata* species complex, monitoring tools have been developed and optimized in nursery systems, particularly in the southern United States (Oliver et al. 2004, Perkovich et al. 2022, 2023, Baker 2024). *Chrysobothri femorata* (Olivier) and related buprestids are attracted to purple-colored traps with a near-infrared peak reflectance (∼740 nm) (Imrei et al. 2020, Perkovich et al. 2022). Rudolph (2022) studied multiple color prism traps (light blue, dark green, and bright red) with or without attractants targeting *C. femorata* and *C. mali* in hazelnut orchards in Oregon. The results were inconclusive due to low pest pressure in the orchard. *Chrysobothris* species are known to be attracted to tree-like shapes (Brooks 1922, Burke and Boving 1929), and the effectiveness of a trap can be enhanced when exposed to direct sunlight (McCullough and Poland 2017, Imrei et al. 2020).

With the reemergence of *C. mali* as a significant pest of walnut trees in California, detailed information on effective monitoring tools and management strategies is warranted. Therefore, in this study, we evaluated various trap types and colors, as well as different lures, to assess their effectiveness in attracting *Chrysobothris mali* in commercial walnut orchards in California.

## Materials and Methods

**Fig. 1.**
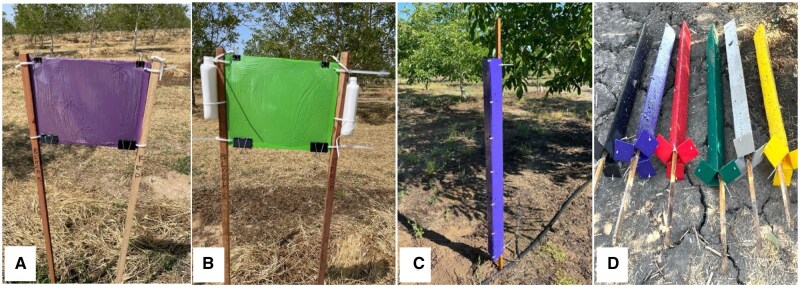
Trap types used in the monitoring of *C. mali* experiments. (A) Unbaited purple panel trap, (B) green panel trap paired with 2× ethanol lure, (C) unbaited purple triangular trap, and (D) different colors of triangular trap.

To identify potential attractive trap colors, types, and lures (Fig. 1) for *C. mali*, monitoring studies were conducted between 2022 and 2024 in commercial walnut orchards in the northern San Joaquin Valley region of California’s Central Valley. Lure and trap type experiments were conducted in a walnut orchard located in Merced County in 2022 and 2023, respectively. The triangular trap color experiments were conducted in 2 walnut orchard sites located in San Joaquin County in 2024. All of these orchard sites had a history of flatheaded borer infestations, which were confirmed through visible signs and symptoms of damage, as well as the presence of larvae in damaged branches. Orchards were maintained following the standard walnut production practices in the region (Ramos 1997). For all experiments, traps were installed around the last week of April, and the study continued through September.

### 2022 Experiment: Evaluation of Trap Colors and Lure Types Using Panel Traps

In this experiment, 2 different colors, green and purple panel traps were used. A panel trap (20 × 30 cm) was made out of a single layer of corrugated plastic (CorrugatedPlastics.Net, New Jersey, United States) (Fig. 1A and B). Two wooden stakes (1.5-m height × 2.5 cm side) (Greenes Fence Wood Stake, Home Depot Inc., Atlanta, GA) were installed on the ground between 2 trees within the row, maintaining a height of 1.2 m (ie 4 ft.) above ground. A clear, double-sided sticky panel (20 × 30 cm) (Trece Inc, Adair, Oklahoma) was affixed to each side of the panel using a stapler gun, and the panel was zip-tied to the top of the 2 ground-installed stakes for stability. The panel traps were then paired with 3 different lure types. Treatments were (i) purple panel with 2 (2×) Trece Ethanol lures, (ii) green panel with 2 (2×) Trece Ethanol lures, (iii) purple panel with 2 (2×) AgBio Ethanol lures, (iv) green panel with 2 (2×) AgBio Ethanol lures, (v) purple panel with (Z)-3-hexenyl acetate lure, and (vi) purple panel with (Z)-3-hexenol lure. Ethanol lures were commercially available from Trece and AgBio (AgBio Inc., Westminster, Colorado) as high-dose ethanol lures. The Ethanol gel matrix was enclosed in collapsible plastic tubes. A single Trece high-release ethanol released 900 to 1,400 mg ethanol/d, while the AgBio single high-release ethanol tube lure released 1,000 mg/d. These release rates were predetermined for products commercially available at the time of the study.

Green leaf volatiles from stressed trees attacked by *C. femorata* were previously identified (Perkovich et al. 2024) and formulated into lures. (Z)-3-hexenyl acetate (95% pure) and (Z)-3-hexenol (98% pure) stock chemicals sourced from Millipore Sigma (EMD Millipore Corporation, Burlington, Massachusetts, United States) were used to prepare the lures in the lab. Briefly, 100 μl of the compound was placed in a 400 μl polypropylene snap cao microcentrifuge tubes (BioPlas, Inc., San Rafael, California). These lures permeated the plastic and volatilized at a rate of approximately 1.7 mg/day for (Z)-3-hexenyl acetate, and 0.01 mg/day, at 30 °C. Release rates were determined by holding the lures in a heat block (*n* = 4) in a fume hood for 7 d (Karla Addesso, unpublished data). Because summer temperatures in California’s northern San Joaquin Valley can reach up to 40 °C, the release rate may have varied under field conditions. As the lures lasted for 4 wk, the estimated release rate is approximately 2 to 5 mg/day, depending on temperature and lure type. Six treatments were replicated 4 times using every third tree row (∼22.8 m) as a treatment row, and treatments were 5 trees apart (30.5 m) within the row. Six treatments were randomized within each treatment row (ie block). Traps were installed at the end of April and checked weekly, starting the first week of May and continuing through the end of September. The lures were changed at 4-wk intervals. All buprestids were recorded and collected for identification. The representative beetle samples from each week’s collections were sent to Dr. Addesso’s Lab at Tennessee State University for identification confirmation (Nelson et al. 1981, Arnett et al. 2002, Wellso and Manley 2007, Nelson et al. 2008).

### 2023 Experiment: Comparison Between Panel and Triangular Traps

A new trap type, the purple triangular trap, was evaluated alongside the purple panel trap (Fig. 1). Purple panel traps (20 × 30 cm, described in the earlier subsection) were installed as previously described. The experiment was conducted in a walnut orchard with 5 treatments and 4 replications. Five treatments evaluated were (i) purple panel with (Z)-3-hexenol lure, (ii) purple panel with (Z)-3-hexenyl acetate lure, (iii) purple panel with 2× ethanol (Trece) lure, (iv) unbaited purple panel, and (v) unbaited purple triangular trap. The triangular trap was included based on the success of similar traps for *C. femorata* on the East Coast (Perkovich et al. 2023). The triangular trap (1.2 m tall × 10 cm wide) was installed vertically from the ground, and its outer surface was coated with TAD Insect Trap Coating (Great Lakes IPM, Vestaburg, Michigan). Treatments were randomized within 2 trap rows (north and south sides of the block), with a 30.8 m buffer between replication blocks. Traps were spaced 4 trees apart (approximately 22 m). Lures were replaced every 4 wk to maintain continued efficacy.

### 2024 Experiment: Evaluations of Trap Colors Using Triangular Traps

In 2024, a different trap color evaluation study (Fig. 1D) was conducted in 2 orchards in San Joaquin County. Since unbaited triangular traps captured high numbers of *C. mali* in 2023 trials compared to the panel traps, 6 different colors of unbaited triangular traps were evaluated for attractiveness. Each of the green, red, yellow, black, gray, and purple colored traps was arranged in a completely randomized block design. Each trap color was replicated 4 times at 2 sites, hereafter referred to as Location 1 and Location 2, using the same experimental setup. The design details of these 1.2 m tall triangular traps are described in the methods under the 2023 Experiment sub-section. Six treatments were spaced 5 trees apart (∼27.5 m) within each row, while replications were separated by a 3-row buffer (18.3 m).

### Beetle Sample Collection and Processing

In each trial, all buprestid beetles captured in the traps were carefully removed using forceps and subsequently placed in a labeled plastic zip bag, and the date of collection was recorded. The specimens were then stored in a freezer at 20 °C until they were cleaned. Glue was removed from specimens by immersing the beetles in a histological clearing agent (ie mixture of alkyl hydrocarbons and essential oils), Histo-Clear II (National Diagnostics, Atlanta, Georgia), for 30 to 40 min to dissolve adhesive residues, followed by a subsequent immersion in dishwashing soap to eliminate any remaining Histo-Clear II. Finally, the cleaned specimens were transferred to ethanol, and representative specimens were sent to the Addesso lab at Tennessee State University for identification.

### Statistical Analyses

In 2022 and 2023, *C. mali* adult capture data were collected weekly, summarized, and the best-performing trap type data were presented as line graphs to illustrate the seasonal phenology of adult activity over 2 consecutive years. For comparing trap types in 2022 and 2023 data, the seasonal total number of beetle captures in each trap type were subjected to the Chi-square Goodness of Fit test. For 2024 data, weekly *C. mali* captures from different trap types were summarized and presented as line graphs to illustrate the seasonal activity of the beetles by trap type. For statistical analysis, the total number of *C. mali* captured in each trap type was averaged to obtain the seasonal mean catch per trap, and these means were subjected to ANOVA to compare trap performance. The statistically significant means were further separated using the Tukey-Kramer HSD test. Statistical analyses were performed using JMP Pro. Version 17 (SAS Institute Inc., Cary, North Carolina).

## Results

### 2022 Experiment: Performance of Trap Colors and Lures Using Panel Traps

Results from the 2022 experiment showed that purple panel sticky traps with lure captured the greatest number of adults (χ^2^ = 37.36, df = 5, *P* = <0.001) (Table 1). These panel traps were paired with different lures. The greatest number of beetles was captured in purple panel trap baited with (Z)-3-hexenyl acetate lure, followed by (Z)-3-hexenol lure and 2× ethanol lure.

**Table 1.** Seasonal total number of *C. mali* adults captured during the season in different trap and lure combinations, Merced County, CA

2022 trial	
Lure/Trap type	Total no. of *C. mali*
**Green panel trap with 2× Ethanol Trece lure**	0
**Purple panel trap with 2× Ethanol AgBio lure**	1
**Purple panel trap with 2× Ethanol Trece lure**	4
**Green panel trap with 2× Ethanol AgBio lure**	1
**Purple panel trap with (Z)-3-Hexenyl acetate lure**	15
**purple panel trap with (Z)-3-Hexenol lure**	12
**χ² = 37.36, df = 5, *P* = <0.001**	

2023 trial	
Lure/Trap type	Total no. of *C. mali*
**Purple panel trap with (Z)-3-Hexenol lure**	4
**Purple panel trap with (Z)-3-Hexenyl Acetate lure**	6
**Purple panel trap with 2× Ethanol Trece lure**	2
**Unbaited purple panel trap**	3
**Unbaited purple triangular trap**	23
**χ² = 40.15, df = 4, *P* = <0.001**	

### 2023 Experiment: Comparison Between Panel and Triangular Traps

During the 2023 season, the greatest number of beetles (*n* = 23) was captured in an unbaited purple triangular trap. Compared to the unbaited purple triangular trap, significantly fewer numbers of beetles were captured in unbaited purple panel traps (3 beetles) or purple panel trap baited with (Z)-3-hexenol (4 beetles), or (Z)-3-hexanyl acetate (6 beetles), or 2x ethanol (2 beetles).

### 2024 Experiment: Effect of Different Color Triangular Traps

**Fig. 2.**
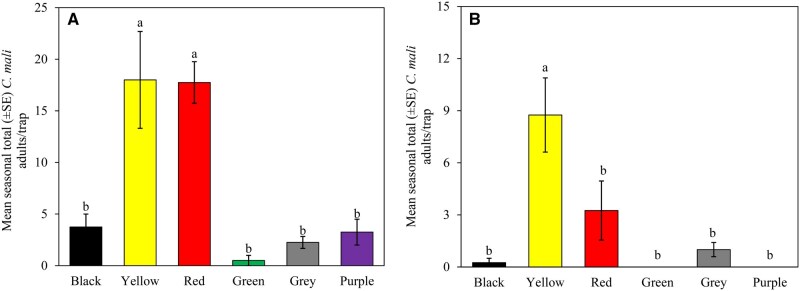
Average seasonal total number of *C. mali* beetles captured in different colors of unbaited triangular traps in walnut orchards at (A) Location 1 and (B) Location 2, San Joaquin County, CA, during the 2024 season. Different letters above bars indicate significant differences in mean numbers captured, as determined by Tukey’s HSD (*P* < 0.05, *N* = 24).

Results from the Location 1 trial showed that unbaited yellow and red triangular traps captured significantly more adult beetles than any other colors (*F *= 18.13, df = 5, 18, *P *< 0.0001) (Fig. 2A). Purple trap capture was not statistically different from green, black, or gray traps. In Location 2, yellow trap outperformed other colors (*F *= 12.26, df = 5, 18, *P *< 0.001) (Fig. 2B). There was not a significant difference in captures between red, grey, and black traps (Fig. 2B). No beetles were captured in purple and green traps throughout the season.

**Fig. 3.**
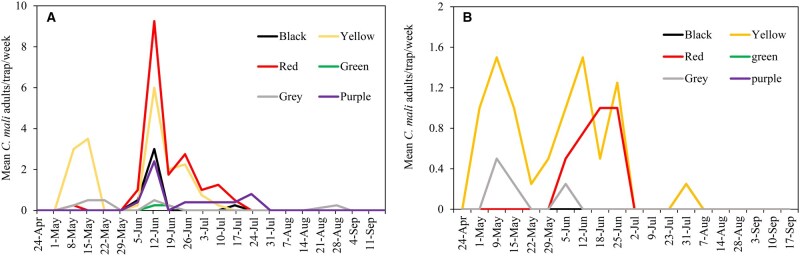
Weekly average number of *C. mali* beetles captured in unbaited triangular traps in walnut orchards at (A) Location 1 and (B) Location 2, San Joaquin County, CA, during the 2024 season.

While assessing the seasonal activity of *C mali* adults, yellow triangular traps captured more beetles in the early part of the season (ie the first 3 wk of May) in both orchards (Fig. 3A and B). Although the red trap captured the greatest number of adults in Location 1 and Location 2 combined, the yellow trap still appeared to be more consistent in capturing the beetle throughout the season, especially in the early part of the flight. The peak capture was recorded in the second week of June in Location 2 and the third week of June in Location 1 (Fig. 3A and B).

## Discussion

*Chrysobothris* species are common pests of fruit, shade, and nut trees in the United States. The rise of *Chrysobothris* infestations in nursery and tree crops across the United States is a growing problem, making the need for tools to monitor and manage these pests more critical than ever. Trap color plays a significant role in eliciting responses in many buprestids, with green and purple being particularly effective in attracting the highest number of emerald ash borer, *Agrilus planipennis* (Francese et al. 2005, Crook et al. 2009, Francese et al. 2010). In this study, we investigated the effectiveness of various trap designs, colors, and lures for monitoring the Pacific flatheaded borer, *Chrysobothris mali*, in California walnut orchards.

The trapping results from 2022 and 2023 provide a preliminary baseline for *C. mali* adult seasonal activity in California walnut orchards (Supplementary Fig. S1A–C), though these data must be interpreted within the context of ongoing trap optimization. Adult emergence began in early May in 2022 and 2024 but was delayed by 2 wk in 2023 (Fig. 3A and B, Supplementary Fig. S1A–C) due to cooler spring and early summer temperatures. Specifically, May and June 2023 maximums were 3 to 5 and 7 to 9 °C lower than in 2022 and 2024, respectively (Supplementary Fig. S2). Peak captures shifted accordingly, highlighting that these phenological shifts are critical for timing summer insecticide applications against *C. mali* (J.P.R., unpublished data).

Our findings indicate that purple panel trap capture more beetles than green panel trap, regardless of the lure type used in the 2022 trials. Other studies have concluded that the most effective trap color for certain buprestid species, particularly *C. femorata*, falls within the violet range (300 to 400 nm) of the electromagnetic spectrum (Cavaletto et al. 2020, Perkovich et al. 2022). (Z)-3-hexenyl acetate and (Z)-3-hexenol as attractants in purple panel traps attracted the highest number of beetles, followed by 2× ethanol in the purple panel traps in our experiment. Several studies have examined how the green leaf volatile component, hexenol, improves the trapping of emerald ash borer in forest systems (Crook et al. 2014). However, the total seasonal capture rates in our trials using these volatiles do not seem conclusive enough to support their usefulness for *C. mali* monitoring in walnut orchards.

Using trap shapes that mimic the tree trunks of the preferred tree hosts has been shown to capture higher numbers of *Chrysobothris* in other trapping studies (Perkovich et al. 2022, 2023, Baker 2024). The purple triangular-shaped traps captured over 37 *Chrysobothris* and 60 buprestid beetles in a season at an ornamental tree nursery (Perkovich et al. 2023). In our 2023 experiment, the ground-installed 1.2 m tall purple triangular trap (10 cm on each side) without any lure outperformed all the other panel traps, whether with or without lures. However, the performance of the purple triangular trap in the presence of a lure was not evaluated in this study. Similar to our results, the best trap design for monitoring *C. femorata* was purple plastic trap folded into a triangular shape (Perkovich et al. 2023). Although triangular-shaped traps are more common, any trap design that mimics tree trunks or branches might be attractive to *Chrysobothris* beetles. Baker (2024) reported good capture of *Chrysobothris* species using 1.52 m tall round poles with diameters ranging from 1.27 cm to 7.62 cm, with higher capture rates in smaller traps (ie <2.54 cm diameter).

Different trap colors for buprestid beetles have been studied in North America, especially following the invasion of *A. planipennis* in 2002 (Haack et al. 2013, Coleman et al. 2014, Petrice and Haack 2015, Rutledge 2020, Kuhn et al. 2024). Research on the influence of trap color in capturing bark- and wood-boring beetles suggests that green and black traps are generally the most effective (Cavaletto et al. 2020). In Italy, trap color had a greater impact on the ambrosia beetle (Curculionidae) capture rates than on bark beetle (Coleoptera: Scolytidae), with purple traps proving more attractive than green ones (Marchioro et al. 2020). Another study indicated that green multi-funnel traps baited with multi-lure and positioned in the canopy could be an efficient trapping strategy for a cerambicid beetle, *Hylotrupes bajulus* and many buprestid beetles associated with broadleaf trees (Rassati et al. 2019).

Oliver et al. (2004) examined multiple color traps in nursery trees and found that a young tree silhouette trap in red color is the most attractive for buprestids, including *C. femorata*. Perkovich et al. (2022) examined multiple trap colors, and their findings indicated that buprestids preferred purple, with a peak reflectance of 405 nm. At the same time, species in the genus *Chrysobothris*, including those in the *C. femorata* species complex, were also attracted to magenta (670 nm) and medium pink (660 nm). Several *Agrilus* species and other metallic-colored buprestids were also primarily drawn to green and purple traps (Cavaletto et al. 2020). In a multi-colored trapping experiment, *Chrysobothris* and buprestids at the family level strongly preferred red traps with a peak reflectance of 700 nm over traps of other colors (Perkovich et al. 2022). Yellow fluorescent traps performed significantly better than the green traps for buprestid beetles in studies conducted in Belgium and France (Kuhn et al. 2024). These findings align with our results, which show that yellow is the most attractive color, followed by red. In buprestids, species-specific color preference can be a result of visual cues associated with their feeding or oviposition hosts. Several *Chrysobothris* prefer to oviposit on the sunny side of the nursery trees (Oliver et al. 2010, Seagraves et al. 2013, Dawadi et al. 2019). Similarly, high levels of damage from *C. mali* have been observed on the sunny sides of branches in California walnut orchards (J.P.R., unpublished data). Many buprestid beetles rely heavily on visual cues to locate suitable hosts, often responding to reflectance in the longer wavelengths of the visible spectrum (yellow to red, ∼580 to 670 nm). These wavelengths are commonly associated with heat-exposed or stressed tree bark (Prokopy and Owens 1983, Crook et al. 2009, Francese et al. 2010), which reflects strongly in these spectral regions, making these surfaces more visually conspicuous against surrounding foliage (Campbell and Borden 2005). Kuhn et al. (2024) suggested that the attraction of several species of *Agrilu*s spp to yellow or green traps may be due to their resemblance to host tree foliage. The relatively higher attraction to yellow traps observed in our study may also indicate an association between this color and stressed or declining trees. *Chrysobothris mali* is known to be highly attracted to stressed trees.

Limited research has been conducted on trapping *C. mali* in commercial walnuts in California. Rijal and Seybold (2019) tested purple Lindgren funnel traps, green prism traps, and purple prism traps in California walnut orchards and found that purple funnel trap was the most effective in attracting *C. mali* compared to green funnel trap. However, in our current study, we tested more colors and found that purple is not the best candidate for *C. mali*, unlike *C. femorata* complex (Perkovich et al. 2022), which is abundant in the southeastern United States. This could be due to the relatively distant lineage between these 2 species (Westcott 1990). Other *Chrysobothris* species also exhibited differences in attractiveness to various colors, ranging from red sticky traps for *C. affinis* (Varandi et al. 2018) to black and green funnel traps for *C.* rugosiceps (Westcott et al. 2018).

Choosing the optimal trap color for the target species is crucial for enhancing early detection efforts (Rassati et al. 2019, Cavaletto et al. 2020, Mrchioro et al. 2020). In our study, yellow traps began capturing beetles at least 1 to 2 wk earlier than red, purple, and black color traps. Thus, our findings confirm that yellow traps are the most reliable option for the early detection of emerging adults. This is particularly important for planning effective pest management strategies throughout the season.

Our current study found that yellow and red triangular traps are effective in capturing *C. mali*. The findings of this study have the potential to enhance pest monitoring and management programs for *C. mali* in walnut orchards by improving capture rates and contributing to more effective monitoring of pest populations. The design and color found effective in this study can be used by pest control advisors and growers to detect and monitor *C. mali* in walnut orchards. The triangular traps used in this study were custom-made. Flat corrugated plastic sheets can be purchased from commercial suppliers (eg CorrugatedPlastics.Net, New Jersey, United States). If the sheet is larger, cut a piece measuring 1.2 m high by 30 cm wide to make one trap. Fold the sheet lengthwise, creating 10 cm flaps on each side to form a triangular shape. Making a shallow internal cut along the fold lines will help the sheet bend cleanly. Secure the open edges with 3 small zip ties to maintain the triangular structure. The outer surface of the trap can then be coated with TAD Insect Trap Coating (Great Lakes IPM, Vestaburg, Michigan) to create a sticky surface for insect capture.

Since these traps are not commercially available, it can be challenging for growers to use them for large production areas. The need for applying glue, the time spent installing the traps, and their susceptibility to dust and debris hinder their practicality. Future research should aim to develop commercially available, more user-friendly versions of these traps. Additionally, future research will explore the potential to improve their effectiveness by integrating new attractants to achieve synergistic effects.

Effective pest monitoring tools are a prerequisite for successful IPM programs. While a visual survey for borer damage signs can be used to monitor this pest in orchards, those signs may not become apparent until the pest is already established or substantial damage has occurred. Therefore, the trap-and-lure combination found to be effective in this study can serve as a pre-detection tool for flatheaded borers in walnut orchards. Moreover, an effective insect monitoring trap can provide critical information such as relative pest population levels and flight activity, and inform the need for and timing of control interventions. Currently, insect counts from these traps provide information on relative pest pressure within an orchard. With further research on the relationship between trap captures and damage, these traps could be used to develop treatment thresholds and serve as a management decision-support tool.

## Supplementary Material

nvag033_Supplementary_Data
