# Supplementary material for: Optimizing trap design, lure, and color for monitoring Chrysobothris mali (Coleoptera: Buprestidae) in California walnut orchards
Source: Environ Entomol. 2026 Apr 16;55(2):nvag033. doi: 10.1093/ee/nvag033 (PMC13107124; doi:10.1093/ee/nvag033)
Supplement: nvag033_Supplementary_Data [file nvag033_supplementary_data.zip › Supp Fig 1a-c.pptx]

## Slide 1
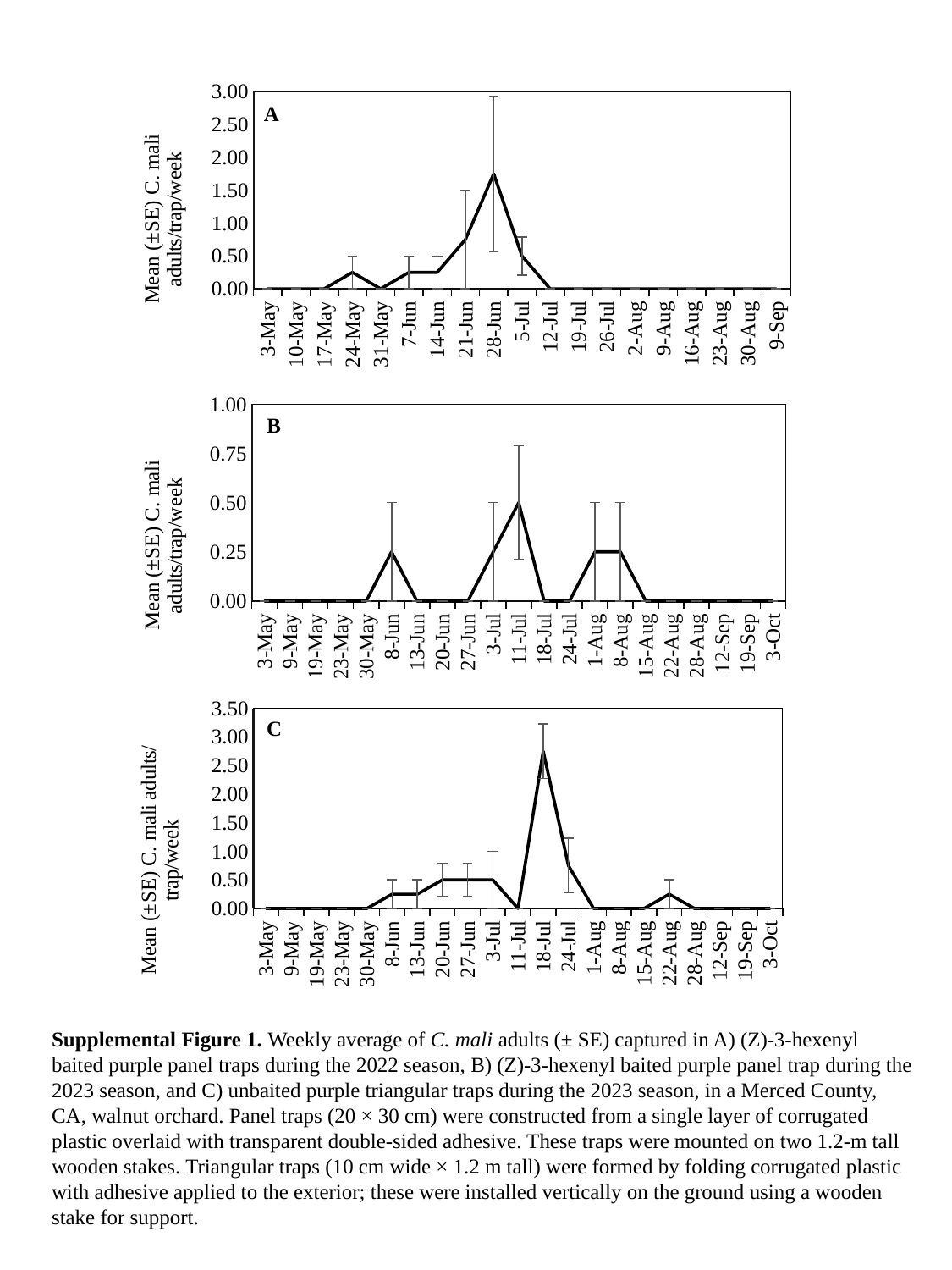

### Chart
| Category | |
|---|---|
| 44684 | 0.0 |
| 44691 | 0.0 |
| 44698 | 0.0 |
| 44705 | 0.25 |
| 44712 | 0.0 |
| 44719 | 0.25 |
| 44726 | 0.25 |
| 44733 | 0.75 |
| 44740 | 1.75 |
| 44747 | 0.5 |
| 44754 | 0.0 |
| 44761 | 0.0 |
| 44768 | 0.0 |
| 44775 | 0.0 |
| 44782 | 0.0 |
| 44789 | 0.0 |
| 44796 | 0.0 |
| 44803 | 0.0 |
| 44813 | 0.0 |A
### Chart
| Category | |
|---|---|
| 45049 | 0.0 |
| 45055 | 0.0 |
| 45065 | 0.0 |
| 45069 | 0.0 |
| 45076 | 0.0 |
| 45085 | 0.25 |
| 45090 | 0.0 |
| 45097 | 0.0 |
| 45104 | 0.0 |
| 45110 | 0.25 |
| 45118 | 0.5 |
| 45125 | 0.0 |
| 45131 | 0.0 |
| 45139 | 0.25 |
| 45146 | 0.25 |
| 45153 | 0.0 |
| 45160 | 0.0 |
| 45166 | 0.0 |
| 45181 | 0.0 |
| 45188 | 0.0 |
| 45202 | 0.0 |B
### Chart
| Category | |
|---|---|
| 45049 | 0.0 |
| 45055 | 0.0 |
| 45065 | 0.0 |
| 45069 | 0.0 |
| 45076 | 0.0 |
| 45085 | 0.25 |
| 45090 | 0.25 |
| 45097 | 0.5 |
| 45104 | 0.5 |
| 45110 | 0.5 |
| 45118 | 0.0 |
| 45125 | 2.75 |
| 45131 | 0.75 |
| 45139 | 0.0 |
| 45146 | 0.0 |
| 45153 | 0.0 |
| 45160 | 0.25 |
| 45166 | 0.0 |
| 45181 | 0.0 |
| 45188 | 0.0 |
| 45202 | 0.0 |C
Supplemental Figure 1. Weekly average of C. mali adults (± SE) captured in A) (Z)-3-hexenyl baited purple panel traps during the 2022 season, B) (Z)-3-hexenyl baited purple panel trap during the 2023 season, and C) unbaited purple triangular traps during the 2023 season, in a Merced County, CA, walnut orchard. Panel traps (20 × 30 cm) were constructed from a single layer of corrugated plastic overlaid with transparent double-sided adhesive. These traps were mounted on two 1.2-m tall wooden stakes. Triangular traps (10 cm wide × 1.2 m tall) were formed by folding corrugated plastic with adhesive applied to the exterior; these were installed vertically on the ground using a wooden stake for support.
