# Supplementary material for: Optimizing trap design, lure, and color for monitoring Chrysobothris mali (Coleoptera: Buprestidae) in California walnut orchards
Source: Environ Entomol. 2026 Apr 16;55(2):nvag033. doi: 10.1093/ee/nvag033 (PMC13107124; doi:10.1093/ee/nvag033)
Supplement: nvag033_Supplementary_Data [file nvag033_supplementary_data.zip › Supp Fig 2.pptx]

## Slide 1
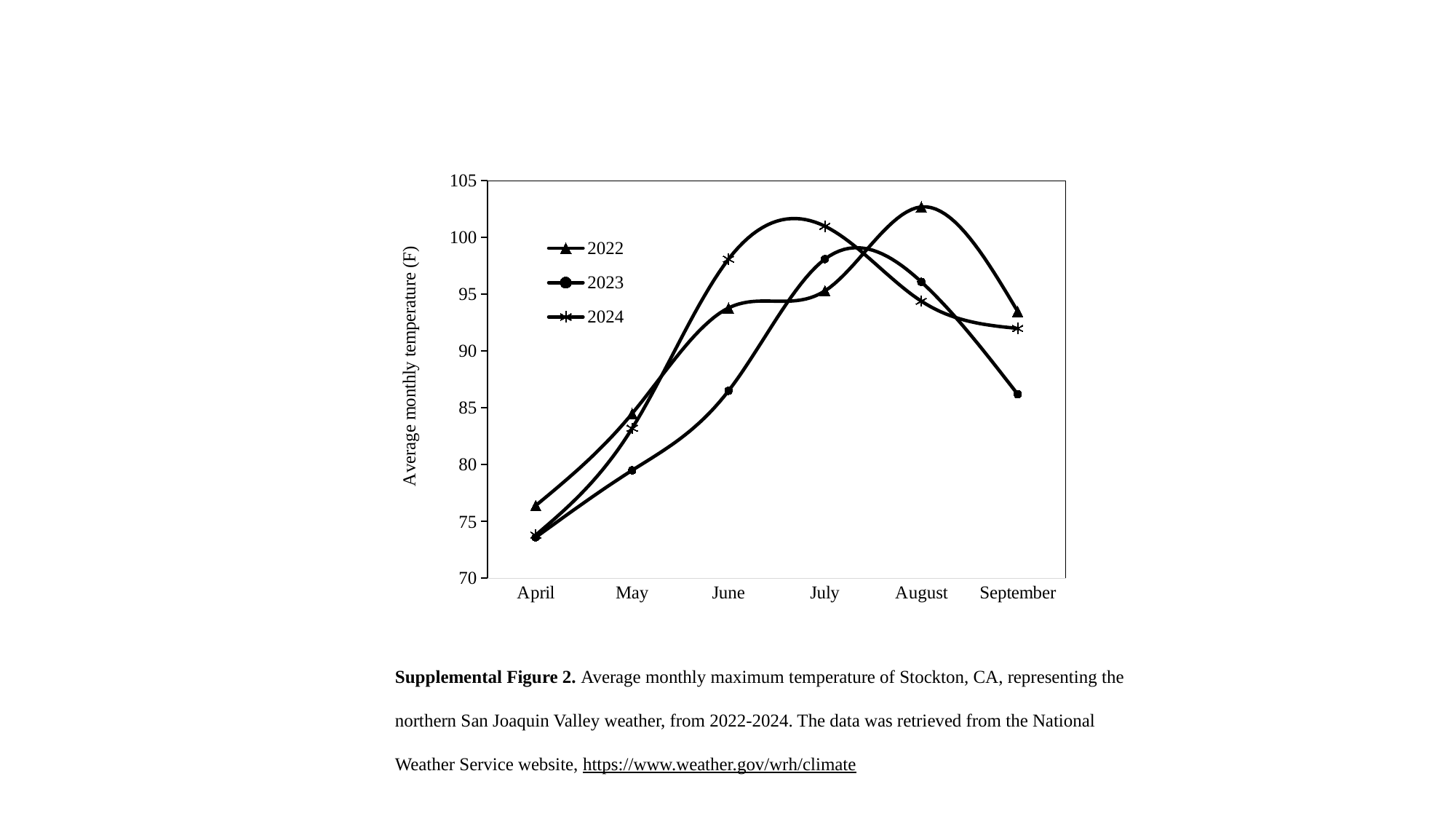

### Chart
| Category | 2022 | 2023 | 2024 |
|---|---|---|---|
| April | 76.4 | 73.6 | 73.8 |
| May | 84.5 | 79.5 | 83.2 |
| June | 93.8 | 86.5 | 98.1 |
| July | 95.3 | 98.1 | 101.0 |
| August | 102.7 | 96.1 | 94.4 |
| September | 93.5 | 86.2 | 92.0 |Supplemental Figure 2. Average monthly maximum temperature of Stockton, CA, representing the northern San Joaquin Valley weather, from 2022-2024. The data was retrieved from the National Weather Service website, https://www.weather.gov/wrh/climate
